# Trends and Factors Associated with Healthcare Utilization for Childhood Diarrhea and Fever in Ethiopia: Further Analysis of the Demographic and Health Surveys from 2000 to 2016

**DOI:** 10.1155/2020/8076259

**Published:** 2020-02-18

**Authors:** Berhanu Teshome Woldeamanuel

**Affiliations:** Department of Statistics, College of Natural Sciences, Salale University, Fiche, Oromia, Ethiopia

## Abstract

**Background:**

Healthcare use for childhood illness reduces the risk of under-five deaths from common preventable diseases. However, rates of healthcare seeking for childhood diarrhea and fever remain low in most low- and middle-income countries including Ethiopia. This study aimed to assess the trends and factors for healthcare diarrhea and fever in Ethiopia from 2000 to 2016.

**Methods:**

Analysis of healthcare use for diarrhea and fever trends was done using data from four Ethiopian Demographic Health Surveys. Descriptive statistics were used to report sample characteristics and healthcare use for diarrhea and fever trends, and chi-square tests were used to assess associations between independent variables and healthcare utilization in each survey. Binary logistic regression analysis was fitted to find the factors related to healthcare utilization for diarrhea and fever. All variables with odds ratio *p* values <0.05 were considered as significant determinants of the outcome.

**Results:**

Healthcare seeking for diarrheal illness significantly increased from 13% (95% CI: 12.5–13.5) in 2000 to 44% (95% CI: 43.2–44.78) in 2016, while healthcare uses for fever significantly increased from 22% (95% CI: 16.7–27.3) in 2000 to 35% (95% CI: 34.3–35.7) in 2016. Factors of healthcare seeking for diarrhea in 2000–2016 were as follows: maternal age <30 years, urban residence, being a male child, nonexposure to mass media and not hearing information about oral rehydration, no desire to have more children, poor wealth index, and region. Meanwhile, factors for healthcare seeking for fever in 2000–2016 were as follows: a long distance from the nearest health facilities, first birth order, nonexposure to mass media, no desire to have more children, maternal age <30 years, urban residence, region, absence of antenatal and postnatal care utilization, poor wealth index, and being born from uneducated mothers (*p* values <0.05 were considered as significant determinants of the outcome.

**Conclusions:**

Factors associated with healthcare utilization for diarrhea and fever differed between 2000 and 2016. Though Ethiopia has achieved a significant reduction in under-five mortality, it needs to accelerate the reduction through strengthening healthcare utilization for common childhood illness to avoid deaths from preventable diseases.

## 1. Background

Worldwide, the number of under-five deaths fell from 12.7 million to 5.9 million deaths between 1990 and 2016, which means every day 16,000 children die under the age of 5 compared with 35,000 deaths in 1990, whereas neonatal mortalities changed from 5.1 million deaths in 1990 to 2.7 million in 2015 [1, 2]. The global under-five death rate fell by 53% between 1990 and 2016; also, the annual rate of reduction increased from 1.8% in 1990–2000 to 3.9 percent in 2000–2015 [3]. Most child deaths were from sub-Saharan Africa (SSA), with an annual reduction rate of only 1.6% in 1990–2000 and 4.1% in 2000–2015.

Globally, about 6.3 million children and young adolescent deaths occurred in 2017 and most, about 5.4 million, deaths in the world were from preventable causes [4]. The sustainable development goal (SDG) indicator, 3.2 ensures to end preventable deaths of newborns and under-five aiming to reduce under-five deaths to at least lower than 25 per 1000 live births by the year 2030 [5]. The UN Interagency Groups in 2015 reported that out of 1995 countries, only 62 countries (24 with low and middle incomes) met the millennium development goal (MDG) 4 with targets of reducing under-five mortality rates by two-thirds between 1990 and 2015 [1]. Despite this progress, in 2017, the universal under-five death rates have been declining to 39 deaths per 1000 live births [2]. Many countries in SSA, including Ethiopia, need to speed up the progress of reducing the high child deaths worldwide. According to the UN Interagency Group's report in 2017, the average under-five mortality rate in the SSA region was 76 deaths per 1,000 live births. This event means that everyone among 13 children was dying before celebrating his or her fifth birthday. The mortality rate in sub-Saharan Africa is 14 times higher than the average ratio of 1 in 185 in developed countries and 20 times higher than the ratio of one in 263 in Australia and New Zealand, which has the lowest regional under-five mortality rate [4].

However, the trend estimates developed by the UN Interagency Group for child mortality in 2018 showed that about 56 million children under 5 years of age are expected to die between 2018 and 2030, with half of them being newborns. If the 50 countries that have under-five mortality above the SDG of 25 deaths per 1000 live births in 2017 accelerated the decline of under-five deaths and achieved the SDG target 3.2.1 and 3.2.2 by 2030, about 10 million lives of children under-five could be saved [4].

According to the Institute for Health Metrics and Evaluation (IHME), global burden of disease estimate in 2017, the highest diarrheal death rates were seen in sub-Saharan Africa and South Asia, ranging from 50 to 150 per 100, 000, where the highest rate of 150 per 100, 000 was observed in the Central African Republic.

In 2015, the global world health statistics reported that the most important causes of under-five mortalities were preterm birth complications (17%), pneumonia (15%), birth asphyxia (11%), diarrhea (9%), malaria (7%), congenital anomalies (7%), and neonatal infections (15%). Removal of these preventable child deaths requires information about healthcare service utilization on the major causes of deaths [6, 7]. In Ethiopia, UNICEF global data reported pneumonia (20.8%), diarrhea (15%), intrapartum (12.3%), preterm (9.6%), and meningitis (7.6%) as the leading cause of deaths among children in 2000. In a similar study in the period 2000–2017, UNICEF reported that diarrhea accounts for a higher percentage (14.9%) as cause of postneonatal mortality in Ethiopia. For the last 5 years, 2012–2017, diarrheal diseases were the first cause of childhood mortality, which accounts for the deaths of 32,875 children per annum on average in Ethiopia. Generally, in Ethiopia, pneumonia (27.3%) and diarrhea (22.6%) were the main causes of postneonatal mortality in 1990–2017 [2].

According to Ethiopia Demographic and Health Survey (EDHS) 2016 report, the prevalence of under-five, infant, and neonatal mortalities were 67, 48, and 29 deaths per 1000 live births, respectively. Geographically, Afar (125 deaths per 1000 live births), Benishangul-Gumuz (98 deaths per 1000 live births), Somali (94 deaths per 1000 live births), and Dire Dawa (93 deaths per 1000 live births) were regions in Ethiopia with the highest under-five mortality, while Addis Ababa has the lowest under-five mortality rate (39 deaths per 1000 live births) [8]. Compared to the global estimate in 2017, all regions except Addis Ababa have an extremely high under-five mortality rate.

The rate of healthcare use for common childhood illnesses in developing countries is low mostly in sub-Saharan Africa. For instance, the United States Agency for International Development report showed that treatment seeking in Eastern Africa countries was in the range of 14%–72% for diarrhea and 18%–100% for fever, with a significant increase over the period 2000–2015 [9]. In Ethiopia, only 13% were seeking treatment for diarrhea in 2000 and only 22% were seeking treatment for fever. After fifteen years in 2016, the percentage of children who sought treatment for diarrhea and fever increased to 44% and 35%, respectively [8, 10].

Ethiopia has introduced the health extension program in 2003 targeted at improving accessibility and provision of most healthcare services to save the life of the newborn and reduce maternal mortalities through healthcare service's utilization [11, 12]. Since then, the country has made a progressive decline in under-five mortality rates to achieve the MDG 4 target. In Ethiopia, only 38.5% of children aged 12–23 months, born five years before the 2016 survey, had received all basic vaccinations, while 44% and 35% of children under age five reporting diarrhea and fever in the last two weeks before the survey were seeking treatment from a health facility. To achieve significant reductions in child mortality to the SDGs in Ethiopia, it is important to consider the progress on the reduction of child mortalities in the past years and the main causes of child deaths. Prior studies in Ethiopia were mostly focused on identifying factors for healthcare seeking for diarrhea and fever childhood illness; they ignore trends in the utilization of healthcare services for childhood illness and its contribution to under-five deaths towards the achievement of the SDG by 2030. Thus, this study aimed to address this evidence gap in investigating the trend and factors associated with healthcare utilization for childhood diarrhea and fever in Ethiopia between 2000 and 2016.

## 2. Methods

### 2.1. Data Sources and Sample

This study used countrywide representative datasets from the Ethiopian Demographic and Health Surveys (EDHS) for 2000, 2005, 2011, and 2016. The data was obtained from the DHS MEASURE Program with permission [13]. Detailed information about the survey settings and sampling, data sources, collection, retrieval processes, and other details of the survey had been described in the final reports of 2000–2016 EDHS [8, 10, 14, 15]. The main aim of these surveys is to deliver detailed information on fertility, family planning, infant, child, adult, and maternal mortality, maternal and child health, nutrition and knowledge of HIV/AIDS, and other sexually transmitted infections to policymakers and planners. The current study analyzes data from children aged less than five years who were born five years before each survey.

### 2.2. Study Variables and Measures

The two outcome variables of the study were seeking healthcare for childhood diarrhea and fever. The EDHS assessed healthcare seeking for childhood illness by asking women whether the child had diarrhea or fever in the past two weeks before the survey and whether they seek advice or treatment for diarrhea and/or fever illness from health facilities (hospitals, health centers, private clinics, or health posts). Responses were categorized into “yes” if a child sought treatment from the health facilities and “no” if a child did not seek treatment for childhood diarrhea or fever.

The explanatory variables included in this study were reviewed from prior published studies on factors for healthcare utilization for common childhood illnesses. The independent variables were grouped into child characteristics, parental characteristics, and household-related characteristics. The child-related characteristics were the sex of a child, the age of a child, childbirth order, place of delivery, and baby postnatal checkup. Parental characteristics included age of mother, mother's and partner's education level, marital status of mothers, antenatal care attendance, place of residence, and geographical region, while household characteristics incorporated total under-five children, wealth index, and exposure to mass media.

### 2.3. Statistical Analysis

Frequencies and percentages were used to report sample characteristics and healthcare utilization for childhood diarrhea and fever trends, and chi-square tests were used to assess associations between independent variables and healthcare utilization in each survey. Logistic regression analysis was used to estimate the effect of each child, parental, and household-related variables on healthcare utilization for childhood diarrhea and fever in each survey (odds ratios with their 95% confidence intervals). All covariates with *p* value <0.25 in the bivariate analysis were included in the multivariable logistic regression analysis, and adjusted odds ratios were reported with 95% confidence intervals. Multicollinearity between explanatory variables was checked using the variance inflation factor (VIF), VIF values greater than 10 were reported as the existence of multicollinearity, and the fitted models were checked using the likelihood ratio test (LRT) in each survey.

## 3. Results

### 3.1. Descriptive Statistics

Table 1 presents the child-, parent-, and household-related characteristics in the study. The proportion of male and female children is almost uniform (about 50%) in all surveys. The percentage of illiterate mothers decreased from 78% in 2000 to 58% in 2016, and mothers with secondary or higher education increased from 9% in 2000 to 15% in 2016. Similarly, the percentage of mothers who were exposed to mass media increased from 12% in 2000 to 30.9% in 2016. The proportion of mothers with information about the use of oral rehydration salt slightly increased from 71.5% in 2000 to 77% in 2016, while the percentage of mothers who do not have any antenatal follow-up during pregnancy decreased from 67.4% in 2000 to 34.7% in 2016, and mothers who have at least four antenatal visits increased from 15.9% in 2000 to 35.8% in 2016. The proportion of deliveries in health facilities increased from 10.9% in 2000 to 38.5% in 2016. Similarly, baby postnatal checkup increased from 2.6% in 2005 to 19% in 2011 and then fall to 9.1% in 2016.

**Figure 1:**
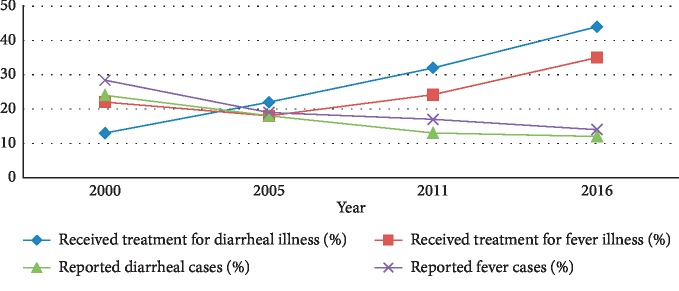
Trends in diarrheal cases, fever cases, and healthcare treatments for diarrhea and fever illness in Ethiopia, 2000–2016. Source: Ethiopian Demographic and Health Survey Report: 2000–2016.

Ethiopia experienced a decline in the percentage of diarrhea and fever illness from 2000 to 2016. There was a significant increase in the proportion of healthcare seeking for diarrheal illness between 2000 and 2011 and then a decrease in 2016. As reported by mothers, the percentage of diarrhea illness among children born five years before the surveys fell by half from 24% in 2000 to 12% in 2016. Similarly, reported cases of fever illness were decreased from 28.4% in 2000 to 14% in 2016. Among the 24% of under-five children who had diarrhea in the 2000 survey, only 13% sought treatment, while of the 12%, only 44% received treatment at health facilities in 2016. Regarding fever illness, only 28.4% of under-five births before the survey had received treatment for fever in 2000, and only 22% had received treatment for fever in 2016. The percentage of seeking treatment for diarrheal illness increased significantly from 13% (95% CI: 12.5–13.5) in 2000 to 44% (95% CI: 43.2–44.78) in 2016, whereas the percentage of seeking treatment for fever increased significantly from 22% (95% CI: 16.7–27.3) in 2000 to 35% (95% CI: 34.3–35.7) in 2016 (Figure 1).

### 3.2. Healthcare Seeking for Childhood Diarrhea Trends in Ethiopia from 2000 to 2016

Table 2 presented healthcare-seeking rates for childhood diarrhea. In all surveys, the healthcare seeking for diarrheal illness among male children was higher than that among females, and healthcare seeking rates were higher among rural residents than urban counterparts. Healthcare seeking rates for childhood diarrhea illness were significantly associated with sex of the child (2000); childbirth order and total number of under-five births (2000, 2011, and 2016); mother's age, mother's education, partner's education, information about use of oral rehydration salt, antenatal visits, place of delivery, and geographical region (2000–2016); mother's exposure to mass media (2000, 2005, and 2011); place of residence (2000–2011); wealth index (2005 and 2011); and baby postnatal care in (2005–2016) (Table 2).

### 3.3. Healthcare Seeking for Childhood Fever Illness Trends in Ethiopia from 2000 to 2016

Table 3 presents healthcare-seeking rates for fever by children, mothers, and household characteristics. Children with the fourth and higher birth order, born from younger mothers (less than 30 years of age), born from rich families, whose mother had information on use of oral rehydration salt, and whose parents want to have more children in the future were seeking treatment for diarrheal illness. The healthcare seeking for diarrheal illness rates among rural children increased significantly from 19.8% (95% CI: 17.8–21.8) in 2000 to 31% (95% CI: 28.3–33.7) in 2011. Similarly, the care seeking for a diarrhea illness rate among mothers who heard of oral rehydration use increased from 22.6% (95% CI: 20.3–24.9) in 2000 to 35.4% (95% CI: 32.4–38.4) in 2011. Healthcare seeking for fever also varies with the number of under-five children in households, mother's age, mother's education, husband's education, exposure to mass media, antenatal care visits, place of delivery, place of residence, and the wealth index between 2000 and 2016 (Table 3).

### 3.4. Multivariate Analysis

Logistic regression analysis was used to examine the effect of each predictor variable on seeking healthcare for diarrhea and fever for each survey. All covariates that were found to be significant in the bivariate analysis at 25% were included in the multiple logistic regression models.

### 3.5. Assessment of Goodness of Fit of the Model

The goodness of fit of the fitted model was checked using the likelihood ratio test (LRT), goodness of fit test, while the significance of each independent variable included in the model was tested using the Wald test. Thus, the likelihood ratio test for chi-square distribution provides a *p* value <0.0001 in all surveys, implying there is a significant difference between the fitted model and the model with no explanatory variables. The results of the tests of multicollinearity between predictor variables showed no problem of collinearity.

### 3.6. Factors of Healthcare Seeking for Diarrheal Illness in Ethiopia

The multivariate analysis of factors associated with healthcare seeking for diarrhea was presented in Table 4. It showed that in 2000 healthcare seeking for diarrheal illness was significantly associated with sex of a child, place of residence, mother's age, mother's exposure to mass media, desire for the last child, information about use of oral rehydration salt, and geographical region. The finding revealed that the odds of healthcare seeking for diarrhea were 0.137 times lower among children whose mother was not exposed to mass media compared to children born from mass media-exposed mothers (AOR = 0.14, 95% CI: 0.03–0.67). The odds of seeking healthcare for diarrhea among children born from mothers aged less than 30 years were 40% higher than those from mothers aged 30 and older in 2000 (AOR = 1.40, 95% CI: 1.01–1.94). Children in the urban areas were 2.289 times more likely to seek healthcare for diarrheal illness (AOR = 2.29, 95% CI 1.45–3.62) compared with rural births in 2000, whereas the odds of healthcare seeking for diarrhea among male children were 59% higher (AOR = 1.59, 95%CI: 1.25–2.02) than those among females. Geographically, Amhara, Oromia, Somali, Benishangul-Gumuz, SNNPR, Gambella, Harari, and Dire Dawa had higher odds of healthcare seeking for diarrheal illness compared to Addis Ababa. This event may be due to the few number of diarrheal cases reported in Addis Ababa and mothers in the other regions not getting access to health facility near to their homes.

Similarly, Table 4 showed that in 2005 healthcare seeking for diarrheal illness was significantly associated with mother's age, information about use of oral rehydration salt, region, and wealth index. Children belonging to mothers less than 30 years old had 47% (AOR = 1.47, 95% CI: 1.01–2.13) increased odds of using healthcare for diarrheal illness compared with those belonging to mothers aged 30 and higher. On the other hand, children whose mothers did not have information about oral rehydration salts use had lower odds of seeking healthcare for diarrhea (AOR = 0.25 95% CI: 0.18–0.35). Similarly, children belonging to households with the poorest wealth index had 0.41 times lower odds of seeking healthcare for diarrhea (AOR = 0.41, 95% CI: 0.21–0.77). Concerning regions, the Tigray region had lower odds of healthcare seeking for diarrhea than Addis Ababa (AOR 0.21, 95% CI: 0.05–0.88).

In 2011 survey healthcare seeking for diarrheal illness was significantly associated with mother's exposure to mass media, information about use of oral rehydration salt, region, wealth index, mother's antenatal care visits during pregnancy, and baby postnatal checkup. Table 4 revealed that mass media exposure was inversely significantly associated with healthcare seeking for diarrhea in 2011 survey. The odds of healthcare seeking for diarrhea were 0.61 time lower among children whose mothers were nod exposed to mass media compared to the mass media-exposed mothers (AOR = 0.61, 95% CI: 0.41–0.92). Being the resident of Benishangul-Gumuz region was associated with higher odds of healthcare seeking for diarrhea compared to being residents of Addis Ababa (AOR = 6.73, 95% CI: 1.14–39.63), whereas being born from a poor mother was associated with lower odds of healthcare seeking for diarrhea compared to children belonging to the richest wealth index (AOR = 0.32, 95% CI: 0.16–0.62). Moreover, children born from mothers without information about oral rehydration salt were less likely to seek healthcare for diarrhea (AOR = 0.33; 95% CI: 0.23–0.47) compared to those from mothers with information about oral rehydration salt use. Similarly, absence of postnatal checkup for baby was associated with 0.53 times lower odds of healthcare seeking for diarrhea compared to receiving postnatal checkup in 2011 survey (AOR = 0.53, 95% CI: 0.29–0.97).

On the other hand, the multivariate analysis presented in Table 4 showed that desire for the last child, information about use of oral rehydration salt, region, mother's antenatal care visits during pregnancy, and husband's education level were factors significantly associated with healthcare seeking for diarrhea in 2016 survey. Children from mothers who did not want to have more children were 1.31 times more likely to seek healthcare for diarrhea (AOR = 1.31, 95% CI 1.04–1.65) than those from mothers who want more children. Being residents of the Tigray region (AOR = 1.37; 95% CI: 1.07–2.53), Afar region (AOR = 2.24; 95% CI: 1.17–4.28), and Gambella region (AOR = 1.91; 95% CI: 1.01–3.61) was significantly associated with higher odds of healthcare seeking for diarrhea compared to being residents of Addis Ababa. Children born from mothers with no antenatal care attended during pregnancy were less likely to seek healthcare for diarrhea (AOR = 0.65; 95% CI: 0.47–0.90) in 2016 survey compared with those born from mothers who attended at least four antenatal visits. Similarly, children from mothers who did not have information about oral rehydration salt were 0.53 times less likely to seek healthcare for diarrhea (AOR = 0.53; 95% CI: 0.40–0.70) compared to those from mothers who had information about oral rehydration salt use. Similarly, the odds of healthcare seeking for diarrhea were lower among children whose fathers attended no education (AOR = 0.64, 95% CI: 0.46–0.89) in 2016 (Table 4).

### 3.7. Factors of Healthcare Seeking for Fever Illness in Ethiopia

Table 5 presents a multivariate analysis of healthcare seeking for fever illness. In 2000 survey, healthcare seeking for fever was significantly associated with mother's exposure to mass media, desire to have more child, childbirth order, place of residence, antenatal care visits during pregnancy, and geographical region of residence. The odds of healthcare seeking for fever illness were about 50% higher (AOR = 1.53, 95% CI: 1.08–2.15) among children with the first birth order than those of the fourth and higher birth orders. Children born to mass media-unexposed mothers were 0.113 times less likely (AOR = 0.11, 95% CI: 0.02–0.52) to seek treatment for fever compared to those of mass media-exposed mothers in 2000. The increased odds of healthcare seeking for fever exist among urban residents compared with rural counterparts (AOR = 1.86, 95% CI: 1.30–2.67). Being born from a mother with no desire to have more children had higher odds of healthcare seeking for fever (AOR = 1.32, 95% CI: 1.05–1.66) in 2000. Concerning healthcare seeking for fever by region, Afar, Oromia, Somali, Benishangul-Gumuz, Southern Nations Nationalities and People (SNNPR), Gambella, Harari, and Dire Dawa regions had higher odds of seeking healthcare for fever compared to Addis Ababa in 2000 survey.

In 2005 survey, children belonging to mothers less than 30 years old with no maternal education at all, and with no postnatal checkup had decreased odds of healthcare seeking for fever illness (AOR = 0.61, 95% CI: 0.40–0.93; AOR = 0.31, 95% CI: 0.12–0.80; and AOR = 0.28, 95% CI: 0.14–0.56, resp.). A long distance from health facilities was also associated with lower odds of healthcare seeking for fever (AOR = 0.63, 95% CI: 0.44–0.90) compared with cases where distance is not a big problem. Similarly, children of first birth order were 1.57 times more likely to seek healthcare for fever (AOR = 1.57, 95% CI: 1.00–2.47) than those of the fourth and higher birth orders in 2005. Compared to Addis Ababa, children from the Tigray and Harari regions had lower odds of healthcare seeking for fever. On the other hand, absence of postnatal visits was related to lower odds of healthcare seeking for childhood fever (AOR = 0.28, 95% CI: 0.14–0.56) in 2000 compared to attendance of postnatal visits.

Regarding the 2011 survey, healthcare seeking for fever was significantly associated with childbirth order, wealth index, and antenatal care visits during pregnancy. Table 5 shows that in 2011, the odds of healthcare seeking for fever illness among first birth order children were 1.57 times higher (AOR = 1.57, 95% CI: 1.00–2.47) than those of the fourth and higher birth order. However, children belonging to the poor wealth index with no antenatal visits or with antenatal care visits less than the number recommended (four) by the WHO were inversely associated with the odds of healthcare seeking for childhood fever (AOR 0.47, 95% CI: 0.274–0.804; AOR 0.522, 95% CI: 0.365–0.745; and AOR 0.627, 95% CI: 0.434–0.904, resp.).

Lastly, in 2016 DHS, the odds of healthcare seeking for childhood fever differ by region, place of residence, and mothers' work status. The odds of heath care seeking for fever were 0.77 times lower in children whose mothers were not working (AOR = 0.77, 95% CI: 0.62–0.95) compared to the working group. The higher odds of healthcare seeking for fever existed among urban children compared with rural counterparts (AOR = 1.76, 95% CI: 1.19–2.60), whereas the Tigray and Afar regions had higher odds of healthcare seeking for childhood fever compared to Addis Ababa in 2016 survey (Table 5).

## 4. Discussion

The healthcare use for childhood diarrhea and fever illness rate in Ethiopia is still considered very low, although there was considerable increase from 13% in 2000 to 44% in 2016 for diarrheal illness and from 22% in 2000 to 35% in 2016 for fever treatment. The increase in trends of healthcare use for diarrheal illness was higher compared to the increase in fever treatment seeking. Besides, the decrease in the number of reported diarrheal cases was also slightly lower compared to fever cases between 2000 and 2016. Prior studies also reported a similar trend [16–18]. However, this finding is lower than that of previous studies conducted in Addis Ababa (70.8%), Mekelle (76%), Bangladesh (75.16%), and Gambia (81.5%) [19–22]. The finding of the study also showed that healthcare seeking for fever was lower than that of a study conducted in Ghana [23]. These inconsistent results might be due to the difference in the socioeconomic status of the study participants.

The increase in the healthcare use for common childhood illness rates in Ethiopia in 2000–2016 may be linked to the high interventions government has taken to expand health centers, child vaccination, and health extension workers in the rural areas [24, 25]. However, this slight increase in healthcare utilization between 2000 and 2016 is insufficient compared to other African countries and its contributions to under-five deaths in the country [26–28]. The results revealed that factors related to healthcare use for childhood diarrhea and fever illness in Ethiopia differ from 2000 to 2016. The reason for this may be due to an improvement in the livelihood of the people, expansion of urban and rural to urban migration, expansion of health centers and infrastructure in the rural parts, increased awareness of mothers about the child feeding practices, and maternal and baby healthcare services use during and after delivery [29].

The study found significant associations between seeking healthcare for diarrhea and exposure to mass media, maternal age, place of residence, sex of a child, wealth index, hearing of oral rehydration, maternal education, partner's education, desire to have more children, number of antenatal visits and baby postnatal checkups, and region. Similarly, seeking healthcare for fever was strongly associated with childbirth order, exposure to mass media, desires to have more children, maternal age, maternal education, working status of mothers, region, number of antenatal visits and baby postnatal checkups.

Healthcare seeking for diarrhea was highly probable among mothers who live in urban areas compared with rural counterparts. Children born to urban residents were more likely to seek healthcare for diarrhea than rural births. This finding agrees with findings from other earlier researches [30, 31]. However, place of residence was not significantly associated with seeking healthcare for fever, which corresponds to previous studies in Bangladesh and Ethiopia [17]. In our study, male children were more likely to get healthcare for diarrhea at health facilities as compared to females. This finding agrees with previous studies conducted in Bangladesh [21], Nigeria [32], and Nepal [29], while some researchers found no significant association between seeking healthcare for diarrhea and sex of a child [19].

Mothers who had a big problem of getting medical help for their children are less likely to seek medical treatment for fever. Findings from previous researches in Ghana, Tanzania, and Ethiopia suggest that seeking healthcare for common childhood illness was less likely among mothers who had a big problem of getting medical help for self [20, 23, 33]. Maternal age was another variable identified as a significant predictor of seeking healthcare for diarrhea and fever in the multivariate analysis. The findings show that children born from younger mothers whose age at birth is below 30 years have significantly higher odds of seeking healthcare for diarrhea and were strongly associated with higher odds of seeking healthcare for fever compared to those born from mothers whose age is 30 years and higher. This finding was consistent with the result of most studies [9, 16, 17, 21] that younger mothers had a high likelihood of seeking healthcare at health facilities.

This study also found that the poor household wealth index has decreased rates of healthcare use to diarrhea, which was in line with previous studies [17]. This event may be due to the fact that poor households do not have financial access. Mothers' educational level was another variable found to be significantly associated with healthcare seeking for fever. The rates of seeking healthcare for diarrhea or fever were inversely associated with the mothers' educational level. Mothers with no education or with primary education level had lower odds of seeking healthcare for childhood illness compared to mothers of secondary or higher education. This finding was congruent with the results of prior studies in low-middle-income countries [9, 17, 21, 23, 34, 35]. The illiteracy of the husband/partner was also directly associated with seeking healthcare for diarrhea, as children that belong to uneducated fathers have higher odds of not seeking healthcare for diarrhea.

Exposure to mass media and information about oral rehydration salt was important to enhance mothers' use of healthcare for diarrheal treatment to children. The higher odds for healthcare seeking diarrhea were among mothers who was exposed to mass media and heard of oral rehydration. This finding was consistent with earlier studies [18, 21, 36, 37]. Also mothers' exposure to mass media was found to increase the rates of seeking healthcare for fever. The higher odds of seeking healthcare for fever existed among mothers who were exposed to mass media, compared to those who were not exposed, keeping all other covariates constant. Previous studies had reported that access to the media was increasing the awareness of mothers to prevent their babies from common childhood illness [16].

Mothers' antenatal healthcare service use and baby postnatal checkup were also important factors that were significantly related to seeking healthcare for fever. It is vital to encourage mothers to use antenatal and postnatal care services in health facilities and get advice from health professionals to improve the health of their newborns. The findings of this study showed that children whose mothers did not attend any antenatal care visits and with no baby postnatal checkup were less likely to seek healthcare for fever than those whose mothers had attended at least four antenatal care visits and had followed baby postnatal checkup at health facilities, respectively. Similarly, utilization of antenatal and postnatal healthcare services was also positively associated with seeking healthcare for fever. Existing literature also indicates that mothers' antenatal and postnatal follow-up provided higher access to get advice from health professionals on seeking healthcare for babies [38].

The findings of the study also revealed that children of mothers who are not currently working and with poor quintiles of wealth index are less likely to seek healthcare for fever. Furthermore, Mao et al.[16] indicated that working mothers have greater rates of seeking healthcare for fever for their children. Poor wealth index of households was also associated with lower odds of seeking healthcare for diarrhea compared with those children born to a mother with the richest wealth quintile. Several studies in the literature reported that wealth index had a positive relationship with seeking healthcare for childhood diarrhea and fever [17]. Regions were significantly associated with seeking healthcare for diarrhea and fever. However, the variation is not consistent with all surveys. This event may be attributed to sociocultural and improved access to infrastructure.

Additionally, birth order and desire to have more children were found to be significant covariates of seeking healthcare for fever. Compared to third or higher birth border, first birth children were more likely to seek healthcare for fever, and children whose mothers/fathers did not want/desire to have more children were more likely to seek healthcare for fever than those whose mothers/fathers want to have more children [17], while in [16] no significant association was found between birth order and desire and seeking healthcare for childhood.

The study did not find a significant association between child age, total under-five children in the household, sex of a child, place of delivery, and partners' education level and seeking healthcare for a fever, which was inconsistent with prior studies on determinants of seeking healthcare for childhood illness [17]. Birth order, child age, total under-five children, place of delivery, and mother's working status were not significantly associated with seeking healthcare for diarrhea between 2000 and 2016 in Ethiopia. However, this finding differs from earlier studies in Ethiopia [17] and Bangladesh [21] that showed that seeking healthcare for diarrhea was more likely among working mothers and a higher number of under-five births.

## 5. Conclusion

Ethiopia showed a growing trend in the healthcare seeking for diarrhea and fever rates from 2000 to 2016. Factors of healthcare seeking for diarrhea and fever also varied over the years. Exposure to mass media, distance to health facilities, place of residence, maternal age, maternal and partner's education, hearing of oral rehydration, birth order, sex, wealth index, mother/caregiver working status, and antenatal or postnatal care services were significant determinants of seeking healthcare for diarrhea and fever. The healthcare seeking rate was lower compared to other low-middle-income countries in the region. Hence, healthcare providers should advise mothers to bring their children to health facilities when they feel any illness. Additionally, health facilities should be expanded throughout the country so that people can get access to facilities near to their homes.

## Figures and Tables

**Table 1:** Descriptive statistics of the samples 2000–2016 EDHS.

Variables	2000		2005		2011		2016	
	*n*	%	*N*	%	*N*	%	*n*	%
Sex of child								
Male	5095	51.3	4732	51.6	5407	50.9	4537	51.8
Female	4846	48.7	4440	48.4	5224	49.1	4225	48.2
Child birth order								
First birth	1660	16.7	1528	16.7	1910	18	1487	17
Second birth	1562	15.7	1437	15.7	1759	16.5	1427	16.3
Third birth	1291	13	1213	13.2	1410	13.3	1242	14.2
Forth birth and above	5428	54.6	4994	54.4	5552	52.2	4606	52.6
Total under-five birth								
One birth	3876	51.2	3608	51.3	4143	50.5	3566	51.3
Two births	3007	39.7	2735	38.9	3111	37.9	2554	36.7
Three and above births	694	9.2	686	9.8	948	11.6	834	12
Mother's age								
Below 30	5228	46.4	4747	46.7	5745	48.2	4638	47.7
30 and higher	6037	53.6	5412	53.3	6175	51.8	5093	52.3
Mother's education								
No education	8817	78.3	7407	72.9	7548	63.3	5640	58
Primary	1458	12.9	1590	15.7	3290	27.6	2676	27.5
Secondary/higher	990	8.8	1162	11.5	1082	9.1	1415	14.6
Husband's education								
No education	7157	63.5	5946	58.5	5905	49.5	4431	45.5
Primary	2200	19.5	2287	22.5	4088	34.3	3054	31.4
Secondary/higher	1908	16.9	1926	18.9	1927	16.1	2246	23.1
Information about oral rehydration salt								
No	3205	28.5	4476	44.4	3335	28.1	2240	23
Yes	8042	71.5	5607	55.6	8549	71.9	7491	77
Wanted last child								
Wanted then	4643	65.2	4479	68.9	5667	74.6	5332	80.7
Wanted later	1183	16.6	1006	15.5	1297	17.1	881	13.3
Wanted no more	1291	18.1	1014	15.6	634	8.3	397	6
Exposure to mass media								
No	9906	88	8546	84.2	9650	81	6599	69.1
Yes	1353	12	1608	15.8	2260	19	2955	30.9
Antenatal care								
No	4801	67.4	4403	67.7	4189	55.1	2292	34.7
1–3	1187	16.7	955	14.7	1731	22.8	1949	29.5
4 and more	1131	15.9	1148	17.6	1683	22.1	236	35.8
Place of delivery								
Home	6344	89.1	5618	86.4	6295	82.9	4097	62
Health facility	774	10.9	882	13.6	1302	17.1	2513	38
Desire to have more children								
Wants no more children	3805	35.3	4186	42.7	4070	35.2	2875	30.4
Wants more children	6970	64.7	5623	57.3	7484	64.8	6593	69.6
Residence								
Rural	8708	77.3	7819	77	8827	74.1	2456	74.8
Urban	2557	22.7	2340	23	3093	25.9	7275	25.2
Region								
Tigray	1074	9.5	974	9.6	1264	10.6	946	9.7
Afar	747	6.6	685	6.7	1081	9.1	857	8.8
Amhara	1677	14.9	1609	15.8	1611	13.5	1112	11.4
Oromia	1863	16.5	1643	16.2	1568	13.2	1312	13.5
Somali	650	5.8	552	5.4	711	6	964	9.9
Ben-Gumuz	813	7.2	708	7	1013	8.5	800	8.2
SNNPR	1439	12.8	1488	14.6	1427	12	1216	12.5
Gambella	762	6.8	611	6	921	7.7	704	7.2
Harari	627	5.6	567	5.6	748	6.3	570	5.9
Dire Dawa	670	5.9	536	5.3	742	6.2	548	6
Addis Ababa	943	8.4	786	7.7	834	7	666	6.8
Wealth index								
Poorest	—	—	2341	23	3080	25.8	2880	29.6
Poorer	—	—	1720	16.9	1916	16.1	1489	15.3
Middle	—	—	1629	16	1774	14.9	1349	13.9
Richest	—	—	1538	15.1	1831	15.4	1303	13.4
Richer	—	—	2931	28.9	3319	27.8	2710	27.8
Postnatal care								
No	—	—	5565	97.4	6062	81	6007	90.9
Yes	—	—	149	2.6	294	19	598	9.1

**Table 2:** Bivariate analysis of healthcare seeking for childhood diarrhea illness in Ethiopia 2000–2016 by child, parental, and household characteristics.

Variables	Care seeking for diarrhea illness												Percentage difference		
	2000			2005			2011			2016			2000–2005	2005–2011	2011–2016
	%	95% CI	Sign.	%	95% CI	Sign.	%	95% CI	Sign.	%	95% CI	Sign.			
Sex of child															
Male	15.9	14.9–16.9	<0.001	14.4	11.8–16.8	0.799	21.1	18–24.1	0.412	3	2.5–3.5	0.562	1.5	−6.7	−5
Female	10.4	9.50–11.3		13	10.4–15.6		19.1	16–22.2		2.6	2.1–3.1		−2.6	−6.1	18.1
Child age															
0–11	—	—		—	—		11.3	8.2–14.4	0.263	1.8	1.2–2.4	<0.001			9.5
12–23	—	—		—	—		14.4	11–17.6		2.1	1.4–2.8				12.3
24+	—	—		—	—		14.3	11–17.5		1.7	1.3–2.1				12.6
Birth order															
1^st^ birth	5.9	4.8–7.0		5	2.1–7.9		8.7	4.9–12.5	<0.001	1.1	0.6–1.6	0.020	0.9	−3.7	7.6
2^nd^ birth	5	3.9–6.1	<0.001	4.6	1.8–7.4	0.114	7.7	4.2–11.2		1.1	0.6–1.6		0.4	−3.1	6.6
3^rd^ birth	3.5	2.5–4.5		4.1	1.4–6.8		5.6	2.4–8.8		0.8	0.3–1.3		−0.6	−1.5	4.8
4^th^ & above	11.8	10.9–12.7		13.7	11.2–16.2		18.3	15.4–21		2.6	2.1–3.1		−1.9	−4.6	15.7
Total under-five birth															
One birth	12.3	11.3–13.3		10.7	8.0–13.4	0.277	18.2	14.9–22	0.004	3.1	2.5–3.7	0.016	1.6	−7.5	15.1
Two births	11.8	10.6–13.0	0.039	12.6	10.1–15.1		16.7	13.7–20		2.9	2.2–3.6		−0.8	−4.1	13.8
3 & above	2.3	1.2–3.4		4	1.3–6.7		5.1	1.9–8.3		0.9	0.2–1.5		−1.7	−1.1	4.2
Mother's age															
<30	16.3	15.3–17.3	<0.001	17.1	14.5–19.7	0.008	25.3	22–28.4	<0.001	3.1	2.6–3.6	<0.001	−0.8	−8.2	22.2
30+	10	7.6–12.4		10.3	7.9–12.7		14.8	11.9–18		2	1.6–2.4		−0.3	−4.5	12.8
Mother's education															
No educa.	19.4	17.4–21.4		18.7	16.4–21.0	<0.001	24.6	21.8–27	<0.001	2.7	2.3–3.1	<0.001	0.7	−5.9	21.9
Primary	4.1	1.5–6.7	<0.001	6.1	3.0–9.2		12.8	9.3–16.3		1.8	1.3–2.3		−2	−6.7	11
Secondary/higher	2.8	0.6–6.3		2.5	0.01–6.2		2.8	0.001–7		0.6	0.1–1.0		0.3	−0.3	2.2
Husband's educa.															
No educa.	14.5	12.5–16.5		13.4	11.1–15.7	<0.001	17	14–19.9	<0.001	1.9	1.5–2.3	0.002	1.1	−3.6	15.1
Primary	7.2	4.7–9.7	<0.001	8.8	6.0–11.6		16.6	13–19.8		1.9	1.4–2.4		−1.6	−7.8	14.7
Secondary/higher	4.6	1.7–7.5		5.2	1.9–8.5		6.5	2.4–10.6		1.3	0.8–1.8		−0.6	−1.3	5.2
Oral rehydration															
No	3.7	2.0–5.4		5.9	4.0–7.8	<0.001	5	2.6–7.4	<0.001	0.7	0.4–1.0	<0.001	−2.2	0.9	4.3
Yes	22.6	20.3–24.9	<0.001	21.7	18.8–24.6		35.4	32–38.4		4.6	4.1–5.1		0.9	−13.7	30.8
Exposure to media															
No	25.5	23.4–27.6	<0.001	23	20.7–25.3	<0.001	37.1	34–39.9	<0.001	4.3	3.9–4.7	0.852	2.5	−14.1	32.8
Yes	0.7	0.03–4.9		4.4	1.0–7.8		8.4	4.6–12.2		0.8	0.3–1.3		−3.7	−4	7.6
Antenatal care															
No	14.8	12.8–16.8		15.5	132–17.8	<0.001	18.4	15.6–21	<0.001	1.6	1.1–2.1	<0.001	−0.7	−2.9	16.8
1–3	5.7	1.5–9.8	<0.001	5.6	2.6–8.5		11.1	7.7–14.5		2.4	1.7–3.1		0.1	−5.5	8.7
4 and more	5.8	0.9–10.7		6.3	2.8–9.8		10.6	6.7–14.5		3.4	2.7–4.1		−0.5	−4.3	7.2
Place of delivery			<0.001			<0.001			<0.001			<0.001			
Home	22.2	20.2–24.2		23	20.7–25.3		32.8	30–35.5		4.1	3.5–4.7		−0.8	−9.8	28.7
Health facil.	4.1	0.8–7.4	0.901	4.5	0.9–8.0		7.4	3.4–11.4		3.5	2.8–4.2		−0.4	−2.9	3.9
Desire more child						0.070			0.306			0.746			
No more	9	6.7–11.3		12	9.3–14.7		12.1	9.0–15.2		1.6	1.1–2.1		−3	−0.1	10.5
Wants more	17.2	15.0–19.4		15.3	12.8–17.8		28	25–30.9		3.6	3.2–4.0		1.9	−12.7	24.4
Residence															
Rural	19.8	17.8–21.8	<0.001	23	20.7–25.3	<0.001	31	28–33.7	<0.001	3.9	3.5–4.3	0.085	−3.2	−8	27.1
Urban	6.5	3.2–9.8		4.4	1.1–7.7		9.1	5.1–13.1		1.1	0.6–1.5		2.1	−4.7	8
Wealth index															
Poorest	—	—		3.7	1.7–5.7	<0.001	9	6.3–11.7	<0.001	1.3	0.9–1.7		−3.7	−5.3	7.7
Poorer	—	—		4.3	1.9–6.7		6.2	3.0–9.4		0.8	0.3–1.3		−4.3	−1.9	5.4
Middle	—	—		6.5	3.7–93		7.6	4.2–11.0		0.8	0.3–1.3	0.063	−6.5	−1.1	6.8
Richest	—	—		5.1	2.3–7.9		8.1	4.7–11.5		0.8	0.3–1.3		−5.1	−3	7.3
Richer	—	—		7.8	4.4–11.2		9.4	5.3–13.5		1.3	0.9–1.7		−7.8	−1.6	8.1
Postnatal care															
No	—	—		24.2	21.8–26.6	0.023	34.6	31.8–37	0.006	6.5	5.9–7.1	0.003	−24.2	−10.4	28.1
Yes	—	—		1.3	0.002–4.9		2.8	0.001–7		1	0.2–1.8		−1.3	−1.5	0.8

**Table 3:** Healthcare seeking for childhood fever illness in Ethiopia 2000–2016 by child, parental, and household characteristics.

Variables	Care seeking for fever illness												Percentage difference		
	2000			2005			2011			2016			2000–2005	2005–2011	2011–2016
	%	95% CI	Sign.	%	95% CI	Sign.	%	95% CI	Sign.	%	95% CI	Sign.			
Sex of child															
Male	14.2	12.5–15.9	0.092	11.2	9.0–13.4	0.246	15.9	13.7–18	.860	3.1	2.6–3.6	0.258	3.0	−4.7	12.8
Female	12	10.3–13.7		9.6	7.5–11.7		15.2	13–17.4		2.6	2.1–3.1		2.4	−5.6	12.6
Child age															0
0–11	—	—		—	—		9.1	6.9–11	0.538	1.9	1.3–2.5	<0.001			7.2
12–23	—	—		—	—		8.9	6.6–11		1.7	1.1–2.3				7.2
24+	—	—		—	—		13.2	11–15.4		2.1	1.7–2.5				11.1
Birth order															0
1^st^ birth	5.8	3.7–7.9	<0.001	4.9	2.4–7.4	<0.001	7.1	4.5–9.7	<0.001	1.2	0.7–1.8	<0.001	0.9	−2.2	5.9
2^nd^ birth	5.3	3.3–7.3		4.0	1.5–6.5		6.0	3.6–8.4		1.2	0.6–1.8		1.3	−2	4.8
3^rd^ birth	3.8	2.0–5.6		3.3	0.9–5.7		5.0	2.7–7.3		0.8	0.3–1.3		0.5	−1.7	4.2
4^th^ & above	11.3	9.7–12.9		8.5	6.6–10.4		13.1	11–15.1		2.5	2.1–3		2.8	−4.6	10.6
Total under-five birth			0.001												
One birth	13.1	11.3–14.9		10.7	8.3–13.1	0.001	15.4	13–17.7	0.003	3.3	2.7–3.9	0.091	2.4	−4.7	12.1
Two births	10.1	8.5–11.7		7.7	5.7–9.7		11.9	9.8–17		2.8	2.2–3.4		2.4	−4.2	9.1
3 & above	3.1	1.2–5.0		2.3	0.1–4.5		3.8	1.6–6		0.9	0.3–1.5		0.8	−1.5	2.9
Mother's age			<0.001											0	
<30	15.5	13.7–17.3		12.5	10.3–14.7	0.190	19.1	16.9–21	0.001	3	2.5–3.5	<0.001	3	−6.6	16.1
30+	10.7	9.1–12.3		8.2	6.1–10.3		12.1	10–14.2		2.1	1.7–2.5		2.5	−3.9	10
Mother's education			<0.001												
No educa.	17	15.5–18.5		11.4	9.6–13.2	<0.001	17.1	15.1–19	<0.001	2.6	2.2–3	0.008	5.6	−5.7	14.5
Primary	5.4	3.2–7.6		5.5	2.9–8.1		10.7	8.2–13		1.7	1.2–2.2		0.1	−5.2	9
Secondary/higher	3.8	1.0–6.6		3.9	0.2–7.6		3.3	0.2–6.4		0.7	0.3–1.1		−0.1	0.6	2.6
Husband's educa.			<0.001			<0.001									
No educa.	13.2	11.7–14.7		8.3	6.5–10.1		12.1	10.1–14	<.001	1.8	1.4–2.2	<0.001	4.9	−3.8	10.3
Primary	6.2	4.4–8.0		6.7	4.4–9.0		12.9	10.6–15		2.1	1.6–2.6		−0.5	−6.2	10.8
Secondary/higher	6.8	4.3–9.3		5.7	2.7–8.7		6.2	3.3–9.1		1.2	.002–2.6		1.1	−0.5	5
Exposure to media															
No	25.5	23.9–27.1	<0.001	16.6	14.6–18.6	<0.001	24.4	17.8–31	0.001	4.2	38–4.6	0.149	8.9	−7.8	20.2
Yes	0.7	2.8–4.2		4.2	1.2–7.2		6.7	4.2–9.2		0.9	0.4–1.4		−3.5	−2.5	5.8
Antenatal care											<.001				
No	13.1	11.614.6	<0.001	9.8	8.0–11.6	<0.001	12.9	11–14.8	<0.001	1.7	1.2–2.2		3.3	−3.1	11.2
1–3	6	4.0–8.0		3.5	1.2–5.8		7.8	5.5–10		2.2	1.6–2.9		2.5	−4.3	5.6
4 and more	7.1	4.7–9.5		7.5	4.2–10.8		10.5	7.6–13.4		3.5	2.8–4.2		−0.4	−3	7
Place of delivery			<0.001												
Home	20.9	19.4–22.4		15.9	14.0–17.8	<0.001	23.5	21.6–25	<0.001	3.6	3.0–4.2	<0.001	5	−7.6	19.9
Health facil.	5.3	2.7–7.9		4.8	1.6–8.0		7.6	4.7–11		3.9	3.1–4.7		0.5	−2.8	3.7
Desire more child			0.006			0.599									
No more	9.7	7.8–11.6		8.4	6.3–10.5		9.9	7.7–12	0.967	1.4	1.0–1.8	0.128	1.3	−1.5	8.5
Wants more	16.4	14.8–18.0		12.3	10.2–14.4		21.2	19–23		3.8	3.3–4.3		4.1	−8.9	17.4
Residence			<0.001												0
Rural	18.6	17.1–20.1		14.5	12.6–16.4	<0.001	21.8	17.5–26	<0.001	3.5	3.1–3.9	0.007	4.1	−7.3	18.3
Urban	7.6	5.1–10.1		6.2	2.8–9.6		9.3	8.0–11		1.5	1.0–2.0		1.4	−3.1	7.8
Wealth index														0	
Poorest	—	—		2.6	0.9–4.3	<0.001	6.6	4.7–8.5	<0.001	1.2	0.8–1.6	0.015		−4	5.4
Poorer	—	—		2.6	0.8–4.4		4.3	2.2–6.4		0.8	0.4–1.0			−1.7	3.5
Middle	—	—		3.7	1.6–5.8		4.4	2.3–6.5		0.7	0.3–1.1			−0.7	3.7
Richest	—	—		3.3	1.2–5.4		5.7	3.3–8.1		0.8	0.3–1.3			−2.4	4.9
Richer	—	—		8.4	5.3–11.5		10.1	7.1–13		1.6	1.1–2.1			−1.7	8.5
Postnatal care															
No	—	—		16.1	14.1–18.1	<0.001	25.2	23.2–27	0.011	6.5	5.9–7.1	0.001		−9.1	18.7
Yes	—	—		1.8	0.01–5.3		2.2	0.01–5		1	0.2–1.8			−0.4	1.2

**Table 4:** Multivariable logistic regression analyses of factors associated with healthcare seeking for diarrheal illness from the 2000–2016 EDHS.

Variables	2000		2005		2011		2016	
	AOR	95% CI	AOR	95% CI	AOR	95% CI	AOR	95% CI
Total under-five								
1	1.35	0.86–2.11	0.72	0.44–1.17	0.74	0.47–1.18	0.87	0.62–1.24
2	1.51	0.99–2.32	0.73	0.48–1.11	0.74	0.49–1.11	0.98	0.71–1.34
3 and above	1.00		1.00		1.00		1.00	
Birth order								
1^st^	1.41	0.90–2.20	1.25	0.72–2.17	1.55	0.91–2.65	0.98	0.68–1.43
2^nd^	1.09	0.73–1.63	1.15	0.72–1.81	1.15	0.75–1.77	1.05	0.76–1.45
3^rd^	0.76	0.51–1.13	0.97	0.62–1.54	1.01	0.66–1.53	0.98	0.72–1.34
4^th^ and above	1.00		1.00		1.00		1.00	
Media exposure								
No	0.14	0.03–0.67^*∗*^	0.86	0.52–1.44	0.61	0.41–0.92^*∗*^	0.87	0.64–1.18
Yes	1.00		1.00		1.00		1.00	
Desire to have more child								
Wants no more children	0.92	0.68–1.24	1.28	0.92–1.78	0.84	0.60–1.17	1.31	1.04–1.65^*∗*^
Wants more children	1.00		1.00		1.00		1.00	
Mother's age								
Less than 30	1.40	1.01–1.94^*∗*^	1.47	1.01–2.13^*∗*^	1.08	0.76–1.52	1.19	0.92–1.53
30 and above	1.00		1.00		1.00		1.00	
Region								
Tigray	1.93	0.73–5.06	0.21	0.05–0.89^*∗*^	2.37	0.99–14.13	1.37	0.74–0.53^*∗*^
Afar	2.43	0.86–7.12	0.23	0.05–1.07	1.92	0.33–11.29	2.24	1.17–4.28^*∗*^
Amhara	3.87	1.55–9.62^*∗*^	0.59	0.14–2.24	2.02	0.34–11.95	1.34	0.70–2.58
Oromia	4.88	2.04–11.69^*∗*^	0.37	0.10–1.45	2.82	0.48–16.57	1.39	0.74–2.60
Somali	15.09	5.83–39.02^*∗*^	0.32	0.07–1.52	2.15	0.36–12.70	1.11	0.57–2.19
Ben-Gumuz	7.78	3.08–19.65^*∗*^	0.56	0.14–2.26	6.73	1.14–39.63^*∗*^	1.206	0.63–2.33
SNNPR	5.32	2.22–12.77^*∗*^	0.31	0.08–1.20	2.34	0.40–13.57	1.63	0.88–3.02
Gambella	6.89	2.78–17.08^*∗*^	0.52	0.12–2.29	5.25	0.87–31.49	1.91	1.01–3.61^*∗*^
Harari	3.76	1.51–9.33^*∗*^	0.25	0.06–1.05	2.74	0.44–17.11	1.24	0.64–2.40
Dire Dawa	3.67	1.43–9.43^*∗*^	0.27	0.06–1.25	1.56	0.23–10.48	1.88	0.99–3.54
Addis Ababa	1.00		1.00		1.00		1.00	
Residence								
Urban	2.29	1.45–3.62^*∗*^	0.55	0.26–1.17	1.58	0.87–2.87	0.95	0.63–1.42
Rural	1.00		1.00		1.00		1.00	
Wealth index								
Poorest	—	—	0.40	0.21–0.77^*∗*^	0.32	0.16–0.62^*∗*^	0.76	0.47–1.22
Poorer	—	—	0.55	0.30–1.01	0.62	0.31–1.23	0.82	0.52–1.32
Middle	—	—	0.81	0.46–1.43	0.70	0.36–1.35	0.99	0.63–1.59
Richest	—	—	0.74	0.43–1.28	0.57	0.30–1.08	1.10	0.70–1.71
Richer	—	—	1.00		1.00		1.00	
Sex of child								
Male	1.59	1.25–2.024^*∗*^	0.98	0.74–1.30	0.84	0.40–1.10	1.11	0.92–1.34
Female	1.00		1.00		1.00		1.00	
Mother's work status								
Not working	0.91	0.70–1.08	0.90	0.65–1.25	1.217	0.91–1.62	0.89	0.71–1.10
Working	1.00		1.00		1.00		1.00	
Antenatal care visits								
No ANC	0.68	0.44–1.04	0.82	0.49–1.36	0.60	0.39–0.94	0.65	0.47–0.90^*∗*^
1–3	0.90	0.58–1.39	0.87	0.50–1.51	0.73	0.47–1.14	0.854	0.68–1.08
4 and above	1.00		1.00		1.00		1.00	
Information about oral rehydration salt								
No	0.49	0.30–0.58^*∗*^	0.25	0.18–0.35^*∗*^	0.33	0.23–0.47^*∗*^	0.53	0.40–0.70^*∗*^
Yes	1.00		1.00		1.00		1.00	
Husband's education								
No education	1.31	0.80–2.15	1.14	0.65–1.99	0.96	0.53–1.74	0.64	0.46–0.89^*∗*^
Primary	1.45	0.90–2.36	1.06	0.61–1.85	0.93	0.52–1.65	0.77	0.58–1.03
Secondary/higher	1.00		1.00		1.00		1.00	
Mother's education								
No education	0.89	0.44–1.79	0.60	0.20–1.82	1.14	0.38–3.44	1.54	0.02–2.34
Primary	0.83	0.42–1.65	0.63	0.20–1.92	0.85	0.29–2.51	1.66	0.15–2.39
Secondary/higher	1.00		1.00		1.00		1.00	
Postnatal care								
No	—	—	0.61	0.28–1.32	0.53	0.29–0.97^*∗*^	0.79	0.58–1.06
Yes	—	—	1.00		1.00		1.00	

^*∗*^Significant *p* value <0.05.

**Table 5:** Multivariable logistic regression analyses of factors associated with healthcare seeking for fever illness from the 2000–2016 EDHS.

Variables	2000		2005		2011		2016	
	AOR	95% CI	AOR	95% CI	AOR	95% CI	AOR	95% CI
Distance								
Big problem	—	—	0.63	0.44–0.90^*∗*^	1.30	0.98–1.73	1.01	0.81–1.24
Not big	—	—	1.00		1.00			
Total under-five birth								
1	0.93	0.68–1.29	0.85	0.50–1.43	0.81	0.55–1.20	0.73	0.51–1.04
2	0.89	0.65–1.21	0.72	0.45–1.18	0.92	0.65–1.31	0.91	0.66–1.24
3 and above	1.00		1.00		1.00		1.00	
Birth order								
1^st^	1.53	1.08–2.15^*∗*^	1.98	1.20–3.27^*∗*^	1.57	1.00–2.47^*∗*^	1.14	0.79–1.66
2^nd^	1.25	0.91–1.70	1.52	0.90–2.59	1.25	0.86–1.84	1.18	0.86–1.63
3^rd^	1.08	0.79–1.47	1.58	0.88–2.86	1.23	0.86–1.76	0.95	0.69–1.30
4^th^ and above	1.00		1.00		1.00		1.00	
Media exposure								
No	0.11	0.02–0.52^*∗*^	1.09	0.64–1.85	1.12	0.79–1.58	0.97	0.72–1.30
Yes	1.00		1.00		1.00		1.00	
Desire to have more children								
Wants no more children	1.32	1.05–1.66^*∗*^	0.98	0.68–1.40	1.12	0.85–1.49	0.98	0.77–1.25
Wants more children	1.00		1.00		1.00		1.00	
Mother's age								
Less than 30	1.12	0.87–1.45	0.61	0.40–0.93^*∗*^	1.07	0.79–1.45	1.12	0.87–1.45
30 and above	1.00		1.00		1.00		1.00	
Region								
Tigray	0.84	0.46–1.54	0.16	0.04–0.61^*∗*^	0.15	0.02–1.50	1.73	1.05–2.87^*∗*^
Afar	2.31	1.23–4.34^*∗*^	0.40	0.09–1.73	0.39	0.04–3.89	2.55	1.47–4.42^*∗*^
Amhara	1.61	0.89–2.90	0.40	0.11–1.48	0.38	0.04–3.74	0.95	0.53–1.70
Oromia	2.00	1.15–3.48^*∗*^	0.61	0.17–2.13	0.56	0.06–5.50	1.57	0.93–2.66
Somali	3.16	1.70–5.85^*∗*^	0.41	0.09–1.87	0.46	0.05–4.54	0.95	0.53–1.73
Ben-Gumuz	4.04	2.22–7.35^*∗*^	0.81	0.21–3.14	1.09	0.11–10.74	0.70	0.38–1.31
SNNPR	2.12	1.21–3.73^*∗*^	0.67	0.19–2.35	0.50	0.05–4.94	1.20	0.71–2.04
Gambella	5.06	2.81–9.13^*∗*^	0.82	0.21–3.21	1.08	0.11–10.69	1.51	0.86–2.63
Harari	2.44	1.32–4.50^*∗*^	0.20	0.04–0.94^*∗*^	0.33	0.03–3.70	0.85	0.47–1.54
Dire Dawa	1.91	1.03–3.55^*∗*^	0.60	0.14–2.60	0.76	0.07–8.22	1.11	0.63–1.94
Addis Ababa	1.00		1.00		1.00		1.00	
Residence								
Urban	1.86	1.30–2.67^*∗*^	1.25	0.65–2.42	1.61	0.98–2.64	1.76	1.19–2.60^*∗*^
Rural	1.00		1.00		1.00		1.00	
Wealth index								
Poorest	—	—	0.62	0.33–1.22	0.47	0.27–0.80^*∗*^	0.90	0.55–1.47
Poorer	—	—	0.73	0.39–1.37	0.64	0.37–1.09	1.13	0.70–1.84
Middle	—	—	0.83	0.46–1.47	0.61	0.36–1.04	1.16	0.71–1.88
Richer	—	—	0.80	0.46–1.37	0.70	0.42–1.16	1.40	0.89–2.20
Richest	—	—	1.00		1.00		1.00	
Sex of child								
Male	1.14	0.95–1.38	1.23	0.90–1.68	0.99	0.79–1.25	1.12	0.92–1.35
Female	1.00		1.00		1.00		1.00	
Mother's work status								
Not working	1.11	0.91–1.36	0.74	0.52–1.05	1.00	0.78–1.28	0.77	0.62–0.95^*∗*^
Working	1.00		1.00		1.00		1.00	
Antenatal care visits								
No ANC	0.64	0.46–0.89^*∗*^	0.62	0.37–1.01	0.52	0.37–0.75^*∗*^	0.84	0.60–1.16
1–3	0.93	0.68–1.29	0.64	0.36–1.11	0.63	0.43–0.91^*∗*^	0.91	0.72–1.16
4 and above	1.00		1.00		1.00		1.00	
Husband's education								
No education	0.89	0.63–1.26	1.14	0.65–1.99	0.98	0.59–1.62	0.91	0.65–1.28
Primary	0.93	0.66–1.30	0.97	0.56–1.67	1.01	0.62–1.63	1.33	0.99–1.79
Secondary/higher	1.00		1.00		1.00		1.00	
Mother's education								
No education	0.85	0.52–1.37	0.31	0.12–0.80^*∗*^	0.94	0.40–2.22	1.49	0.99–2.24
Primary	1.11	0.70–1.76	0.59	0.23–1.52	0.83	0.36–1.92	1.42	0.99–2.02
Secondary/higher	1.00		1.00		1.00		1.00	
Postnatal care								
No	—	—	0.28	0.14–0.56^*∗*^	0.86	0.53–1.37	0.81	0.61–1.09
Yes	—	—	1.00		1.00		1.00	

^*∗*^Significant *p* value < 0.05.

## Data Availability

The data used to support the findings of this study are available from the corresponding author upon request.

## Abbreviations

DHS: Demographic and Health Survey
EDHS: Ethiopia Demographic and Health Survey
IHME: Institute for Health Metrics and Evaluation
LRT: Likelihood ratio test
MDG: Millennium development goal
SDGs: Sustainable development goals
SSA: Sub-Saharan Africa
UNICEF: United Nations Children's Fund
UN: United Nations
VIF: Variance inflation factor
WHO: World Health Organization.

## Additional Points

*Strength and Limitations of the Study*. This study uses a nationally representative survey dataset, which enhances inferences for the entire country level. The major strength was that it used the most recent singleton live births five years before each survey. However, this study used secondary data. The major limitation is that the study was a mother's recall of events that took place for the past five years preceding the survey, which is subject to recall bias.

## References

[1] UNICEF (2015). *Levels & Trends in Child Mortality, Estimates Developed by the UN Inter-agency Group for Child Mortality Estimation*.

[2] UN IGME (2017). *Levels & Trends in Child Mortality, Report 2017, Estimates Developed by the UN Inter-Agency Group for Child Mortality Estimation*.

[3] World Health Organization (2016). *Under Five Mortality, Global Health Observatory Data*.

[4] United Nations Inter-agency Group for Child Mortality Estimation (UN IGME) (2018). *Levels & Trends in Child Mortality, Report 2018, Estimates Developed by the United Nations Inter-agency Group for Child Mortality Estimation*.

[5] United Nations General Assembly (2015). *Transforming Our World, the 2030 Agenda for Sustainable Development*.

[6] WHO (2015). *World Health Statistics, Report*.

[7] Woldeamanuel B. T. (2019). Socioeconomic, demographic, and environmental determinants of under-5 mortality in Ethiopia: evidence from Ethiopian demographic and health survey, 2016. *Child Development Research*.

[8] Central Statistical Agency (CSA) and ICF (2016). *Ethiopia Demographic and Health Survey 2016*.

[9] Bennett A., Eisele T., Keating J., Yukich J. (2015). *Global Trends in Care Seeking and Access to Diagnosis and Treatment of Childhood Illnesses*.

[10] Wakabi W. (2008). Extension workers drive Ethiopia’s primary health care. *The Lancet*.

[11] Koblinsky M., Tain F., Gaym A., Karim A., Carnell M., Tesfaye S. (2010). Responding to the maternal health care challenge: the Ethiopian Health Extension Program. *Ethiopian Journal of Health Development*.

[12] Central Statistical Agency (CSA) and ICF (2000). *Ethiopia Demographic and Health Survey*.

[13] ICF (2016). *The DHS Program: Demographic and Health Surveys*.

[14] Central Statistical Agency (CSA) and ICF (2005). *Ethiopia Demographic and Health Survey 2005*.

[15] Central Statistical Agency (CSA) and ICF (2011). *Ethiopia Demographic and Health Survey 2016*.

[16] Mao B., Lundy Saint, Nit S. (2013). *Factors Associated with Utilization of Health Services for Childhood Diarrhea and Fever in Cambodia: Further Analysis of the Cambodia Demographic and Health Survey*.

[17] Ayalneh A. A., Fetene D. M., Lee T. J. (2017). Inequalities in health care utilization for common childhood illnesses in Ethiopia: evidence from the 2011 Ethiopian Demographic and Health Survey. *International Journal for Equity in Health*.

[18] Carvajal-Vélez L., Amouzou A., Perin J. (2016). Diarrhea management in children under five in sub-Saharan Africa: does the source of care matter? A Countdown analysis. *BMC Public Health*.

[19] Fissehaye T., Damte A., Fantahun A., Gebrekirstos K. (2018). Health care seeking behaviour of mothers towards diarrheal disease of children less than 5 years in Mekelle city, North Ethiopia. *BMC Research Notes*.

[20] Adane M., Mengistie B., Mulat W., Kloos H., Medhin G. (2017). Utilization of health facilities and predictors of health-seeking behavior for under-five children with acute diarrhea in slums of Addis Ababa, Ethiopia: a community-based cross-sectional study. *Journal of Health, Population and Nutrition*.

[21] Sarker A. R., Sultana M., Mahumud R. A., Sheikh N., Van Der Meer R., Morton A. (2016). Prevalence and health care–seeking behavior for childhood diarrheal disease in Bangladesh. *Global Pediatric Health*.

[22] Saha D., Akinsola A., Sharples K. (2013). Health care utilization and attitudes survey: understanding diarrheal disease in rural Gambia. *The American Journal of Tropical Medicine and Hygiene*.

[23] Ansah E. K., Gyapong M., Narh-Bana S., Bart-Plange C., Whitty C. J. M. (2016). Factors influencing choice of care-seeking for acute fever comparing private chemical shops with health centres and hospitals in Ghana: a study using case control methodology. *Malaria Journal*.

[24] Leon N., Sanders D., Van Damme W. (2015). The role of “hidden” community volunteers in community based health service delivery platforms: examples from sub–Saharan Africa. *Glob Health Action*.

[25] UNICEF and Federal Ministry of Health Ethiopia (2013). *Evaluation of Community Management of Acute Malnutrition in Ethiopia*.

[26] World Data Bank (2013). *World Development Indicators*.

[27] Ministry of Health, Family Health Department (2005). *National Strategy for Child Survival in Ethiopia*.

[28] Mehretie Adinew Y., Feleke S. A., Mengesha Z. B., Workie S. B. (2017). Childhood mortality: trends and determinants in Ethiopia from 1990 to 2015—A systematic review. *Advances in Public Health*.

[29] Målqvist M., Singh C., Kc A. (2017). Care seeking for children with fever/cough or diarrhoea in Nepal: equity trends over the last 15 years. *Scandinavian Journal of Public Health*.

[30] Girma M., Gobena T., Medhin G., Gasana J., Roba K. T. (2018). Determinants of childhood diarrhea in West Gojjam, Northwest Ethiopia: a case control study. *Pan African Medical Journal*.

[31] Gedamu G., Kumie A., Haftu D. (2017). Magnitude and associated factors of diarrhea among under five children in farta Wereda, North West Ethiopia. *Quality in Primary Care*.

[32] Aniugbo B. M., King K., Hayes C., John J. A. F. (2017). The socio-demographic determinants of health care seeking behaviour by carers of children with diarrhoea in rural communities in Enugu state, Nigeria. *International Journal of Current Research*.

[33] Kassile T., Lokina R., Mujinja P., Mmbando B. P. (2014). Determinants of delay in care seeking among children under five with fever in Dodoma region, central Tanzania: a cross-sectional study. *Malaria Journal*.

[34] Malhotra N., Upadhyay R. P. (2013). Why are there delays in seeking treatment for childhood diarrhoea in India?. *Acta Paediatrica*.

[35] Nasrin D., Wu Y., Blackwelder W. C. (2013). Health care seeking for childhood diarrhea in developing countries: evidence from seven sites in Africa and Asia. *The American Journal of Tropical Medicine and Hygiene*.

[36] Victora C. G., Bryce J., Fontaine O., Monasch R. (2000). Reducing deaths from diarrhoea through oral rehydration therapy. *Bulletin of the World Health Organization*.

[37] Mengistie B., Berhane Y., Worku A. (2012). Predictors of oral rehydration therapy use among under-five children with diarrhea in Eastern Ethiopia: a community based case control study. *BMC Public Health*.

[38] Azage M., Haile D. (2015). Factors affecting healthcare service utilization of mothers who had children with diarrhea in Ethiopia: evidence from a population based national survey. *International Electronic Journal of Rural and Remote Health, Education Practice and Policy*.

